# Coronavirus disease 2019 (COVID‐19) and individuals with intellectual and developmental disabilities in Nigeria

**DOI:** 10.1002/pa.2601

**Published:** 2021-02-04

**Authors:** Ogochukwu Ann Ijezie, Hilary Izuchukwu Okagbue, Olufemi Adebari Oloyede, Vanessa Heaslip, Philip Davies, Jane Healy

**Affiliations:** ^1^ Faculty of Science and Technology Bournemouth University Poole UK; ^2^ Department of Mathematics, College of Science and Technology Covenant University Ota Ogun State Nigeria; ^3^ Department of Obstetrics and Gynaecology Olabisi Onabanjo University Teaching Hospital Sagamu Ogun State Nigeria; ^4^ Faculty of Health and Social Sciences Bournemouth University Poole UK; ^5^ Faculty of Social Sciences University of Stavanger Stavanger Norway

**Keywords:** COVID‐19, forecasting models, intellectual and developmental disabilities, Nigeria, pandemic

## Abstract

This article chronicles the present situation of coronavirus disease 2019 (COVID‐19) on individuals with intellectual and developmental disabilities (IDD) in Nigeria. A systematic search was conducted on three bibliographic databases: MEDLINE Complete, Web of Science and Scopus, and supplemented with grey literature searches to assess studies on the effect of COVID‐19 on these individuals in Nigeria with data on this group from December 2019 to July 2020. There were no studies found concerning individuals with IDD in Nigeria. This article argues for an urgent call to action by Nigerian policymakers to make data available to help understand the impact of COVID‐19 and to develop and implement appropriate interventions. This article provides steps to support and care for these individuals in Nigeria. Forecasting models are recommended which offer better approaches in yielding accurate predictions and provide valuable decisions in the event of future threats and infectious disease outbreak in Nigeria.

## 1 INTRODUCTION

The World Health Organization (WHO) received notification about the coronavirus disease 2019 (COVID‐19) that emanated from Wuhan city of Hubei Province of China, on December 31, 2019 (World Health Organization, 2020a; Zhu et al., 2020). Studies have focused on the economic and health impacts of the disease (Anderson et al., 2020; Nicola et al., 2020; Pappa et al., 2020; Wang et al., 2020). Globally, it has been acknowledged that COVID‐19 is linked to psychosocial issues such as depression, loneliness, and anxiety among different social strata which include marginalized groups, healthcare providers, those who tested positive for COVID‐19, quarantined individuals, older people, and children (Dubey et al., 2020; Salari et al., 2020). As of August 26, 2020, there has been an exponential increase in the spread of the virus. There were 23,765,805 confirmed cases of COVID‐19 across over 200 countries and territories, with Africa having 1,019,362 confirmed cases and 20,828 deaths (World Health Organization, 2020b).

According to the Nigeria Centre for Disease Control (NCDC), the first case of COVID‐19 in Nigeria occurred on February 27, 2020, when an Italian citizen entered the country on the February 25, 2020, and tested positive to the virus (Nigeria Centre for Disease Control, 2020a). Initially, Lagos and Ogun States and the Federal Capital Territory (FCT) located in Abuja saw an early increase of cases which quickly progressed into the “community transmission” phase, with daily increases in the number of affected individuals who had no travel history (Bamidele & Daniel, 2020).

Following the guidelines stipulated by WHO on lockdown procedures, the Presidential Task Force on COVID‐19 in Nigeria introduced quarantine and lockdown measures to curb the spread of the disease and protect the health of Nigerians (Office of the Secretary to the Government of the Federation, 2020a). During the lockdown, the stringent measures included: a stay‐at‐home order for all Nigerian citizens; a ban on interstate and intertown travel except for essential services such as agricultural produce, petroleum products, and manufactured goods; a ban on domestic and international flights, except for emergencies; closure of worship centres, businesses, offices, and schools (Office of the Secretary to the Government of the Federation, 2020b). Furthermore, the Nigerian government embarked on public broadcasts to inform communities to ensure compliance with the WHO directives such as the self‐isolation for those with symptoms of COVID‐19, mandatory use of face coverings in public, and continuous practice of handwashing/sanitizing (Office of the Secretary to the Government of the Federation, 2020c). Like other countries under the COVID‐19 crisis, the lockdown restrictions in Nigeria were not applicable to healthcare professionals, journalists, pharmacies, supermarkets and shops selling groceries, and some government agencies (Mbah, 2020). The success of the measures was constrained, because of public dissatisfaction with the insufficient provision of basic needs by the government, such as financial support, particularly for those that depended on daily income for survival and those that are low‐paid, inadequate healthcare beds, testing kits, and personal protective equipment (PPE) (Mbah, 2020). To alleviate the impact of the lockdown, the President of Nigeria approved a 3‐month moratorium on all government‐funded loans and the provision of a 2‐month food supply to internally displaced persons (Okwumbu, 2020).

As of August 26, 2020, the NCDC reported 52,800 confirmed cases of COVID‐19 and 1007 deaths in Nigeria (Nigeria Centre for Disease Control, 2020b). However, official figures did not report the proportion of individuals with intellectual and developmental disabilities (IDD) in Nigeria. For countries like the United States of America (USA) and the United Kingdom (UK), public health policies are based on overall population data, including individuals with IDD (Mills et al., 2020; Office for National Statistics, 2020; Turk et al., 2020).

Individuals with IDD such as those with Down syndrome and autism are a vulnerable group of people with ongoing health challenges, co‐morbidities, and psychosocial challenges requiring additional healthcare needs (United Nations, 2020). Studies have shown that there is a high prevalence of comorbidities such as heart disease, respiratory disease, hypertension, diabetes, and visual impairment among individuals with IDD (Cooper et al., 2015; Dunn et al., 2018), which have been linked with poorer outcomes from COVID‐19 (Turk et al., 2020). Apart from the increased susceptibility, the impact of COVID‐19 on these individuals also stems from the emotional burden from combating safety measures, such as shielding themselves and their family members or caregivers (Hassiotis et al., 2020).

Most individuals with IDD require physical proximity to families or caregivers to support any challenges the individual may have in communication or cognitive abilities to enjoy fulfilling, predictable, and manageable daily lives in times like COVID‐19 (Constantino et al., 2020), which is applicable to those in Nigeria. Nevertheless, supporting individuals with IDD infected with COVID‐19 is challenging with difficulties such as tolerating quarantine and understanding the importance of complying with the public health measures like social distancing and handwashing (Courtenay & Perera, 2020). In a similar manner, those without COVID‐19 might experience emotional difficulties, boredom, and anxiety which could lead to behavioral confrontation toward their families or caregivers and the systems they depend on for care and support (Hassiotis et al., 2020). Consequently, it is imperative that these individuals across the lifespan obtain reassurance, clear communication, and support for alleviating the concerns of boredom and anxiety about their health and families or caregivers (Singh et al., 2020).

Given the foregoing, this paper reviews the current state of knowledge regarding COVID‐19 concerning individuals with IDD in Nigeria. The structure of the paper is as follows: the present section introduces the study and presents the aim; the second section summarizes the rationale for focusing on Nigeria; the third section outlines the methods of study; the fourth section discusses the main findings, and the fifth section provides the conclusion and healthcare policy recommendations.

## 2 WHY NIGERIA?

With an estimate of over 200 million citizens across 923,768 km^2^, Nigeria is the most populous country in Africa and often described as the “Giant of Africa” (World Bank, 2019). Nigeria is classified as Africa's largest economy, and it consists of 36 states and the FCT located in Abuja (World Bank, 2019). Currently, Nigeria uses the definition of the American Association on Intellectual and Developmental Disabilities to describe individuals with IDD; however, most of these individuals have been misdiagnosed due to lack of local expertise, insufficient funding, and multicultural diversity of the country (Sango, 2017). After the country's independence in 1960, the state governments managed the social care of individuals with IDD (Obiakor, 1998). Presently, the majority of these individuals receive support and care from families/caregivers, nongovernmental organizations (NGOs), and some are abandoned and left homeless (Nnama‐Okechukwu & Okoye, 2019).

Studies have shown that individuals with IDD generally receive insufficient attention and support within public health research (Krahn et al., 2015). Despite the legislations of the United Nations Convention on the Rights of Persons with Disabilities aimed at upholding the dignities and human rights of disabled people, these individuals are faced with stigmatization that hinders social inclusion and increases inequalities when compared with the general population across the globe (Jansen‐van Vuuren & Aldersey, 2020). This is challenging in Nigeria, where the predominant cultural and religious beliefs are that people are disabled because of either their misdeeds, parental transgressions, witchcraft, cursed from God, or possessed by evil spirits (Ajuwon, 2012; Oloyede, 2011), and as such these individuals are often marginalized and stigmatized.

Before the COVID‐19 pandemic, there were limited records of support and care provided individuals with IDD in Nigeria. Similarly, there is a dearth of literature on the number of individuals with IDD due to the lack of financial resources to conduct research, lack of awareness, and neglect of these individuals and their families by the government and the society in Nigeria (Sango, 2017). Therefore, this is a knowledge gap that needs to be bridged. However, the issues of allocating resources, inadequacy of policy imperatives, and impact of cultural beliefs that promote epidemiological studies in this area are also experienced in various parts of African countries (Njenga, 2009).

COVID‐19 has affected many countries; however, it was estimated that Nigeria would be part of the African countries at highest risk (Gilbert et al., 2020). Therefore, it is pertinent to investigate the impact on individuals with IDD in Nigeria. There has been a call to action to ensure that these individuals are included in COVID‐19 protection, response, and recovery measures (Office of the United Nations High Commissioner for Human Rights, 2020). With this in mind, the cases of individuals with IDD in Nigeria during the COVID‐19 pandemic are been considered.

## 3 METHODS

### 3.1 Search strategy

**FIGURE 1:**
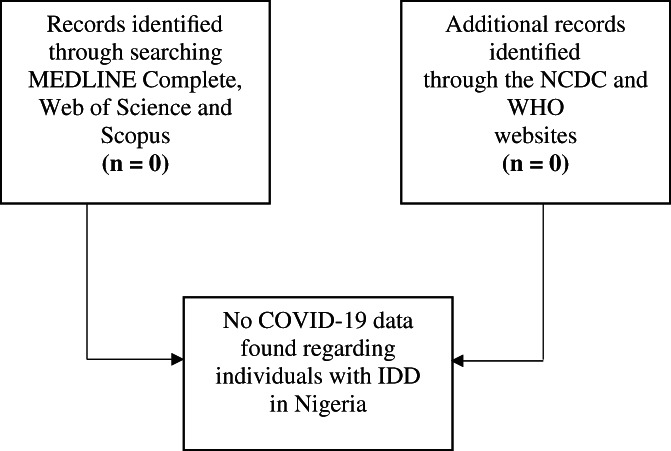
Diagram of searches conducted from December 2019 to July 2020. Source: Created by the Authors based on search on the databases. Note: NCDC = Nigeria Centre for Disease Control, WHO = World Health Organization, IDD = Intellectual and Developmental Disabilities

To assess COVID‐19 publications on individuals with IDD in Nigeria, a systematic search was done on three bibliographic databases (MEDLINE Complete, Web of Science and Scopus) from December 2019 to July 2020. As the WHO was notified about COVID‐19 on December 31, 2019, the search commenced from December 2019. A structured search strategy was developed by combining keywords, Medical Subject Headings (MeSH) terms, titles, and abstracts. The database searches conducted using the keyword combinations: Nigeria*, Africa*, West Africa*, Sub‐Saharan Africa*, Intellectual Disabilit*, Developmental Disabilit*, Learning Disabilit*, Learning Disorder*, Coronavirus diseas*, *COVID‐19* using the Boolean operators (“AND” and “OR” only) (see Appendix S1). It was revealed that no article was found on the database searches concerning individuals with IDD in Nigeria as shown in Figure 1. Grey literature searches were conducted on the NCDC and the WHO websites, and no information was recorded about these individuals in Nigeria where Table 1 shows the epidemiological cases of COVID‐19 in Nigeria.

**TABLE 1:** Epidemiology of COVID‐19 cases in Nigeria as of August 26, 2020

S/N	States affected	Number of cases (laboratory confirmed)	Number of cases (on admission)	Number discharged	Number of deaths
1	Abia	755	105	643	7
2	Adamawa	217	43	159	15
3	Akwa Ibom	271	43	220	8
4	Anambra	202	25	159	18
5	Bauchi	644	85	545	14
6	Bayelsa	378	31	326	21
7	Benue	451	301	141	9
8	Borno	740	58	646	36
9	Cross River	82	11	63	8
10	Delta	1715	151	1518	46
11	Ebonyi	965	17	921	27
12	Edo	2553	201	2252	100
13	Ekiti	235	106	125	4
14	Enugu	1087	214	852	21
15	FCT	5046	3548	1450	48
16	Gombe	714	86	605	23
17	Imo	523	326	187	10
18	Jigawa	322	3	308	11
19	Kaduna	2059	215	1832	12
20	Kano	1721	160	1507	54
21	Katsina	771	290	457	24
22	Kebbi	92	2	82	8
23	Kogi	5	0	3	2
24	Kwara	936	171	740	25
25	Lagos	18,018	2602	15,214	202
26	Nasarawa	421	111	298	12
27	Niger	239	59	168	12
28	Ogun	1631	158	1447	26
29	Ondo	1515	179	1305	31
30	Osun	769	105	648	16
31	Oyo	3058	1289	1732	37
32	Plateau	2185	988	1168	29
33	Rivers	2090	132	1901	57
34	Sokoto	158	4	138	16
35	Taraba	87	9	73	5
36	Yobe	67	0	59	8
37	Zamfara	78	1	72	5
Total		52,800	11,829	39,964	1007

*Note*: FCT = Federal Capital Territory. Source: Nigeria Centre for Disease Control (2020b).

## 4 DISCUSSION OF MAIN FINDINGS

### 4.1 Challenge in obtaining information

Based on the searches conducted on the databases, there is no information in Nigeria regarding how families or caregivers are to support individuals with IDD during COVID‐19 pandemic. Individuals with IDD potentially depend on their families and caregivers for public health guidance such as social distancing rules, respiratory etiquette, handwashing, or other valuable information to minimize the risk of contracting the disease (Brooks et al., 2020). Although, a guidance was provided by the NCDC in collaboration with the Federal Ministry of Health in Nigeria for people classified as “*vulnerable*” and having a high‐risk of contracting COVID‐19 (Nigeria Centre for Disease Control, 2020c), which is provided in Table 2.

**TABLE 2:** Category of people considered as vulnerable in Nigeria

Social strata	Description
Vulnerable group – classified as high risk of contracting COVID‐19	Persons aged 50 years and older (with or without underlying illnesses)
	Persons with critical underlying medical conditions such as diabetes, cancer, lung disease, liver disease, moderate to severe asthma and so forth.
	Other persons who have been assessed as vulnerable, based on clinical assessment

*Note*: Source: Nigeria Centre for Disease Control (2020c).

From Table 2, the guidance shows that there exists a group of persons described as “*vulnerable*” based on clinical assessment; however, it is not clearly stated if individuals with IDD fall into the category. This could be attributed to the stigma affixed to disabled people revealing the extent of social exclusion in the Nigerian society (Etieyibo & Omiegbe, 2016).

### 4.2 Human rights and individuals with IDD

In March 2007, Nigeria signed the United Nations Convention on the Rights of Persons with Disabilities and ratified its Optional Protocol in September 2010, which was an attempt to protect the rights of disabled people (Arimoro, 2019; Umeh & Adeola, 2013). In January 2019, Nigeria finally endorsed the Discrimination Against Persons with Disabilities (Prohibition) Act 2018 subsequent to constant activism by disability rights groups; nevertheless, the country is yet to execute the proper measures in defending the rights of individuals with IDD (Arimoro, 2019). During the COVID‐19 outbreak, a 2‐day virtual conference was organized by the Nigerian government and African NGOs such as the Down Syndrome Foundation Nigeria, Africa Disability Alliance, Africa Down syndrome Network, Cedar Seed Foundation, and Initiative for National Growth Africa and amongst others, to discuss adopting and ratifying the African Disability Protocol (ADP) in all African countries particularly Nigeria (Nweze, 2020). Although, some African countries like the Central African Republic, Rwanda, Togo, South Africa, Gabon, and Burkina Faso have adopted and ratified the ADP.

The ADP is a development designed to improve the quality of life of individuals with IDD in Africa, and it addresses vital issues such as poverty, systemic discrimination, and harmful practices (Nweze, 2020; Obazee, 2020). During the conference, it was added that reports from individuals with IDD in Nigeria could be beneficial by including them in decision‐makings to enable developing an inclusive response and recovery plan from COVID‐19 pandemic (Obazee, 2020). It was highlighted that continuous inclusive technology in Nigeria for these individuals was important due to the unprecedented nature of the challenges posed by the pandemic to enable participation in discussions about them and also for the future (Obazee, 2020).

### 4.3 The healthcare systems and individuals with IDD during COVID‐19

The healthcare sector in Nigeria experiences poor management of facilities and financial difficulties (Adeloye et al., 2017). A major solution is to amend the legislation on National Health Insurance Scheme launched in 2005 so that social health insurance is compulsory and provides financial risk protection to all Nigerians (Onwujekwe et al., 2019). Furthermore, a recent study have suggested that the Nigerian government should improve on the investment in the nation's healthcare sector toward eliminating death‐threatening diseases that leads to low life expectancy in the county such as tuberculosis, pneumonia, and influenza (Alhassan et al., 2020). Following the outbreak of COVID‐19, the healthcare systems were also impacted in Nigeria (Oladele et al., 2020). Similarly, countries like China, Spain, the USA, the UK, Italy, and France experienced major peak in cases which overwhelmed their healthcare systems and additional challenges such as shortage of healthcare workforce capacity to manage the patients, insufficient PPE and ventilators, and inadequate treatment centres (European Centre for Disease Prevention and Control, 2020). In Nigeria, most individuals with IDD experience difficulty in accessing healthcare services and therefore encounter unmet healthcare needs impeding their human rights to basic amenities (Oloyede, 2011). Consequently, this group of people might have encountered this challenge during COVID‐19.

In Nigeria, there is an inadequacy of administrative measures specifically for individuals with IDD and sometimes failure to carry out accurate diagnosis in accordance with the 10th revision of the International Statistical Classification of Diseases and Related Health Problems (ICD‐10) codes (Sango, 2017). Although, the NCDC made commendable efforts to initiating an online course on Infection Prevention and Control to support the general public and healthcare workers to help curtail the risk of spreading COVID‐19 (Nigeria Centre for Disease Control, 2020b). Conversely, there is no recorded data on the healthcare outcomes or support services for individuals with IDD which reveals a lack of adequate structures to monitor COVID‐19 cases among these individuals. This is disturbing as there is a high probability of serious outcomes for this group, such as death from COVID‐19. While this neglect in the public health sector might not be alleviated now, it is important to use all accessible data on individuals with IDD to understand the effects of COVID‐19.

### 4.4 Impact of COVID‐19 on families and caregivers

Amid the global crisis, individuals with IDD received care and support from either families/caregivers or charity organizations (Rose et al., 2020). The COVID‐19 pandemic also added a significant impact upon families and caregivers due to social distancing and isolation measures (Alexander et al., 2020). This might constitute an extra burden especially for those that have relatives with IDD or caregivers with little income and/or having to deal with employment issues in Nigeria. Therefore, the Nigerian government could provide support in meeting the needs of families and caregivers by the provision of food supplies, financial support, and other basic amenities to cushion the effect of the crisis. Furthermore, the Nigerian government could also provide funding to the NGOs that provide care and support to individuals with IDD.

A recent study showed that families or caregivers encounter lots of emotional and psychological stress in supporting their relative with IDD in Nigeria (Chukwu et al., 2019). The reasons are linked to ignorance in society and lack of information, resulting in some of them adopting negative coping strategies such as pretending that the disabled individual does not exist, keeping the disabled person out of public view and locking the disabled person in doors (Chukwu et al., 2019). In unprecedented times like COVID‐19, Nigerian policymakers and stakeholders should develop programmes and services to assist families and caregivers of these individuals to cope as well as protecting their well‐being. These measures would empower them in combating the pandemic. Commentators like Ajuwon (2012) opined that religious values play a vital role in safeguarding most individuals with IDD and their families or caregivers in Nigeria toward minimizing negative impacts of challenges they experience in their daily lives. Specifically, the COVID‐19 pandemic had a multifaceted impact on many human lives, and most families and caregivers may maintain their religious values in Nigeria.

### 4.5 Supporting and protecting individuals with IDD during COVID‐19

**FIGURE 2:**
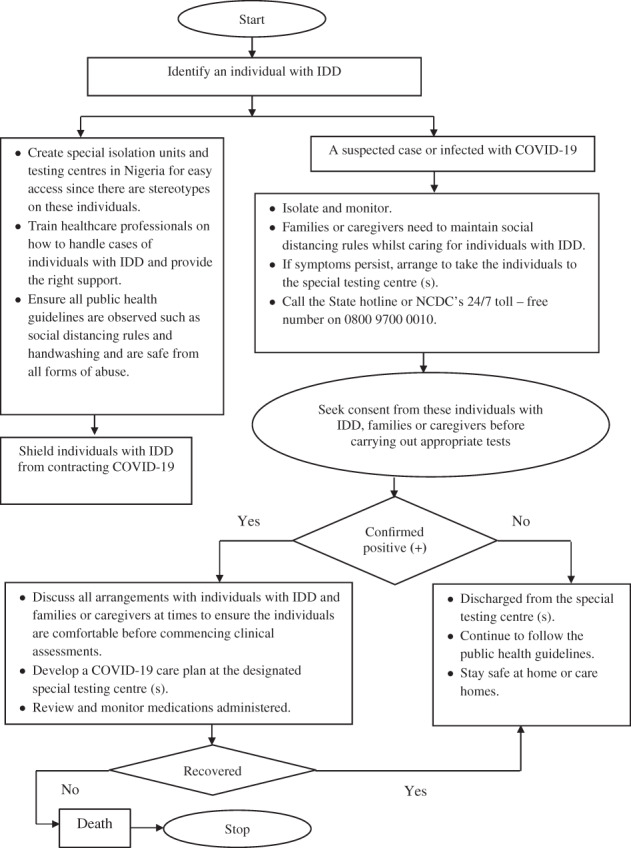
Flowchart on steps to supporting and protecting individuals with intellectual and developmental disabilities from COVID‐19 in Nigeria. Source: Adapted from Alexander et al. (2020). Note: IDD = Intellectual and Developmental Disabilities

During this pandemic, individuals with IDD might find it difficult in advocating for themselves. These individuals are susceptible to neglect and abuse from families and/ or caregivers and the society (Araten‐Bergman & Bigby, 2020). It has been suggested that governments should make special requirements and protect the rights of individuals with IDD in this crisis period across every nation (Clegg, 2020). Therefore, these individuals need to be supported and protected in countries like Nigeria and based on the recommendation of Alexander et al. (2020), some steps have been highlighted (see Figure 2).

In cases where a family member or caregiver of an individual with IDD becomes ill or quarantined, an alternative caregiver who has an experiential knowledge in meeting the needs of the individual with IDD should be provided until a full recovery of the usual family member or caregiver (Jalali et al., 2020). This approach should also be considered by policymakers in Nigeria. Furthermore, similar provisions should be made in the event of losing a family member or caregiver due to COVID‐19.

In this COVID‐19 environment, barriers to accessing healthcare facilities must be eliminated for individuals with IDD, and healthcare workers need to be trained on how these individuals should be protected and supported (United Nations, 2020). The use of technology could also be beneficial in conducting surveys to understand the challenges of individuals with IDD and their families/caregivers encountered during the pandemic; this could be done while observing the social distancing rules and using the appropriate PPE. In the event of future pandemics, robust techniques like forecasting models could be used to generate better strategies and provide valuable decisions (Poletto et al., 2020; Shinde et al., 2020) such as in supporting individuals with IDD.

## 5 CONCLUSION AND HEALTHCARE POLICY RECOMMENDATIONS

The purpose of this review was to gain a better understanding of the present condition of individuals with IDD in Nigeria amid the COVID‐19 pandemic. Following the systematic searches conducted on the bibliographic databases, no results were found in this area including the NCDC and WHO websites.

It is acknowledged that COVID‐19 is a novel coronavirus, and the number of epidemiological cases in Nigeria is not high compared to some developed countries like China, Spain, the USA, the UK, Italy, and France. However, there is an urgent need to obtain data on individuals with IDD in Nigeria for several reasons. First, to know the proportion of individuals that contracted COVID‐19 based on the daily situation reports provided by the NCDC. Second, to investigate the overall well‐being and the level of support received by these individuals during COVID‐19 regarding how they are coping with the effect of the pandemic such as those that have mild to moderate intellectual disabilities living independently in communities; those under parental care and might be dependent on families or caregivers; those that live in care homes or under the care of NGOs/ charity organizations; and those who live in the rural areas and unable to meet their financial needs. Third, to confirm if there are trained healthcare professionals especially handling cases of these individuals. Fourth, to verify if there are special isolation centres designed specifically for these individuals for easy access.

This article demonstrated that the unavailability of data on individuals with IDD in Nigeria is a call to action to Nigerian policymakers to mitigate societal barriers. Suggestions have been provided in Figure 2 on how this group of people could be supported amid the pandemic. Furthermore, an essential step will be the collation and analysis of IDD statistics, which are crucial for monitoring the impact of COVID‐19 at the country level. It is imperative to monitor the trend of the contagious disease among this group paying attention to all ages and genders. In the absence of such statistics, there will be a major deficit in public health policies where individuals with IDD and their families/caregivers will not have standards to assess the degree of effect caused by COVID‐19 and obtain the necessary care and support. The generation of robust statistics like forecasting models will be beneficial in enabling the Nigerian governments to develop strategies in recognizing IDD within generic social and economic policies, as well as proffering solutions in the event of future pandemics to help improve the public health. Some scholars have argued that the lack of reliable and robust statistical data about IDD will incapacitate how governments can efficiently plan and implement services for these individuals (Lang, 2009; Loeb, 2013). This review advocates for the formulation and enforcement of appropriate legislations and policies to provide daily situation reports regarding individuals with IDD and other disabled people in Nigeria in the event of future pandemics. This would promote social inclusion, minimize prejudice, and stigmatization among these individuals and their families and caregivers in Nigeria.

In addition to those already mentioned on individuals with IDD, we recommend data be made available about other at‐risk groups such as people with diabetes, sickle cell anaemia, Alzheimer's disease, and pregnant women so that researchers can investigate the impact of COVID‐19 among these groups.

Drawing upon the statement of the Office of the United Nations High Commissioner for Human Rights (2020), this paper argues that it is pertinent to conduct future work in examining the implication of COVID‐19 among individuals with IDD and their families and caregivers in Nigeria. Based on the evidence in this review, public health authorities in Nigeria, with the support of the country's WHO and United Nations International Children's Emergency Fund (UNICEF) offices, should engage on the provision of funds to conduct intensive research on these individuals. Furthermore, accurate monitoring and accountability of funds at all levels of the Nigerian government (Federal, State and Local) should be provided when the funds are allocated and disbursed for the disease outbreak management; thereby, contributing to global health equity (Omoleke et al., 2018).

Finally, it is the responsibility for all policymakers in Nigeria, scientists, and researchers to recognize and seek the opportunity to offset the burden of COVID‐19 on these individuals alongside their families and caregivers. There is a need to balance public health priorities which must be reflected in practical healthcare development, policy formulation, and implementation in Nigeria (Bakare et al., 2019). Although this research has focused on Nigeria, further investigation of this important area is recommended in other countries.

## CONFLICT OF INTEREST

The authors declare that they have no competing interests.

## ETHICAL STATEMENT

Not applicable.

## Supporting information


**Appendix** **S1.** Supporting information.

## Data Availability

Data sharing is not applicable to this article as no new data were created or analyzed in this study.
