# Supplementary material for: Coronavirus disease 2019 (COVID‐19) and individuals with intellectual and developmental disabilities in Nigeria
Source: J Public Aff. 2021 Feb 4;21(4):e2601. doi: 10.1002/pa.2601 (PMC7995158; doi:10.1002/pa.2601)
Supplement: Supplementary file 1 — Appendix S1. Supporting information. [file PA-21-0-s001.docx]

**Supplementary file:** a) Search strategy

| Bibliographic  databases | # | Search  Queries | Hits |
| --- | --- | --- | --- |
| MEDLINE Complete | 1 | (MH "Nigeria") OR TI "Nigeria*" OR AB "Nigeria*" | 40,453 |
|  | 2 | (MH "Africa+") OR (MH "Africa, Western+") OR (MH" Africa South of the Sahara+") OR TI "Africa*" OR AB "Africa*" OR TI "West Africa*" OR AB "West Africa*" OR TI " Sub-Saharan Africa*" OR AB "Sub-Saharan Africa*" | 409,870 |
|  | **3** | **#1 OR #2** | 419,197 |
|  | 4 | (MH "Intellectual Disability+") OR (MH "Developmental Disability") OR (MH "Learning Disability+") OR (MH "Learning Disorders+") OR TI "Intellectual and Developmental Disabilit*" OR AB "Intellectual and Developmental Disabilit*" OR TI "Intellectual Disabilit*" OR AB "Intellectual Disabilit*" OR TI "Developmental Disabilit*" OR AB "Developmental Disabilit*" OR TI "Learning Disabilit*" OR AB "Learning Disabilit*" OR TI "Learning Disorder*" OR AB "Learning Disorder*" | 147,306 |
|  | 5 | (MH " Coronavirus+") OR TI " Coronavirus diseas*" OR AB " Coronavirus diseas*" OR TI " COVID-19" OR AB " COVID-19" OR TI " Covid-19" OR AB " Covid-19" OR TI " C-19" OR AB " C-19" OR TI " COVID-19 Pandemic" OR AB " COVID-19 Pandemic" OR TI " Covid-19 Pandemic" OR AB " Covid-19 Pandemic" OR TI " C-19 Pandemic" OR AB " C-19 Pandemic" | 43,019 |
|  | **6** | **#3 AND #4 AND #5** | **0** |
| Web of Science | 1 | (TS= ("Nigeria*" OR "Africa*" OR " West Africa*" OR " Sub-Saharan Africa*") )  AND DOCUMENT  TYPES: (Article) | 456,305 |
|  | 2 | (TS = ("Intellectual and Developmental Disabilit*" OR "Intellectual Disabilit*" OR "Developmental Disabilit*" OR "Learning Disabilit*" OR "Learning Disorder*") )  AND DOCUMENT  TYPES: (Article) | 40,154 |
|  | 3 | (TS= ( " Coronavirus diseas*" OR " COVID-19" OR " Covid-19" OR " C-19" OR " COVID-19 Pandemic" OR " Covid-19 Pandemic" OR " C-19 Pandemic") )  AND DOCUMENT  TYPES: (Article) | 14,655 |
|  | **4** | **#1 AND #2 AND #3** | **0** |
| Scopus | 1 | TITLE-ABS-KEY ( "Nigeria*" OR "Africa*" OR  " West Africa*" OR  " Sub-Saharan Africa*" ) | 781,707 |
|  | 2 | TITLE-ABS-KEY ( "Intellectual and Developmental Disabilit*"  OR  "Intellectual Disabilit*"  OR  "Developmental Disabilit*"  OR  "Learning Disabilit*"  OR  "Learning Disorder*" ) | 93,767 |
|  | 3 | TITLE-ABS-KEY ( " Coronavirus diseas*"  OR  " COVID-19"  OR  " Covid-19"  OR  " C-19"  OR  " COVID-19 Pandemic"  OR  " Covid-19 Pandemic"  OR  " C-19 Pandemic" ) | 37,623 |
|  | **4** | **#1 AND #2 AND #3** | **0** |

Note: # = search number for each database conducted separately

b) A collated list of the search

| S/N | Databases | Hits |
| --- | --- | --- |
| 1 | MEDLINE Complete | 0 |
| 2 | Web of Science | 0 |
| 3 | Scopus | 0 |
|  | **Total** | **0** |
